# An Improved Mechanistic-Empirical Creep Model for Unsaturated Soft and Stabilized Soils

**DOI:** 10.3390/ma14154146

**Published:** 2021-07-26

**Authors:** Xunli Jiang, Zhiyi Huang, Xue Luo

**Affiliations:** College of Civil Engineering and Architecture, Zhejiang University, 866 Yuhangtang Road, Hangzhou 310058, China; jxunli@zju.edu.cn (X.J.); hzy@zju.edu.cn (Z.H.)

**Keywords:** soft soil, stabilized soil, rice husk ash, Mechanistic-Empirical creep model, matric suction

## Abstract

Soft soils are usually treated to mitigate their engineering problems, such as excessive deformation, and stabilization is one of most popular treatments. Although there are many creep models to characterize the deformation behaviors of soil, there still exist demands for a balance between model accuracy and practical application. Therefore, this paper aims at developing a Mechanistic-Empirical creep model (MEC) for unsaturated soft and stabilized soils. The model considers the stress dependence and incorporates moisture sensitivity using matric suction and shear strength parameters. This formulation is intended to predict the soil creep deformation under arbitrary water content and arbitrary stress conditions. The results show that the MEC model is in good agreement with the experimental data with very high R-squared values. In addition, the model is compared with the other classical creep models for unsaturated soils. While the classical creep models require a different set of parameters when the water content is changed, the MEC model only needs one set of parameters for different stress levels and moisture conditions, which provides significant facilitation for implementation. Finally, a finite element simulation analysis of subgrade soil foundation is performed for different loading levels and moisture conditions. The MEC model is utilized to predict the creep behavior of subgrade soils. Under the same load and moisture level, the deformation of soft soil is largest, followed by lime soil and RHA–lime-stabilized soil, respectively.

## 1. Introduction

Soft soils are usually regarded to be problematic due to their poor engineering properties, for instance, high water content, low undrained shear strength, poor permeability and remarkable rheological properties [1]. With the increased demand of urbanization, more and more foundations of buildings and infrastructure have been built on soft soil areas [2]. As a result, a series of practical problems have arisen because of insufficient strength and/or excessive deformation of soft foundations and subgrades [3]. In order to mitigate the problems, the following three methods are generally used to treat soft soils [4]: (i) consolidation of soft soil by surcharge preloading; (ii) foundation reinforcement by compaction piles; and (iii) strengthening of soil by chemical stabilization. 

These treatment methods are applicable to different engineering situations. The surcharge preloading method has lower cost but a longer consolidation period, which can be used when the construction period permits. The compaction pile method is usually applicable to sandy soil, loose soil and miscellaneous fill foundation with gravel, brick and rubble. The chemically stabilized method has a wider range of applications because the curing period is relatively short and the effect of stabilization is better controlled. Moreover, compared with the other two methods, the chemically stabilized method is simpler and more economical, so it is one of most popular treatments and has been widely used in engineering practice.

Chemical stabilization is a method of adding a certain amount of curing agent to the soft soil to change the surface properties and connections between the particles through physical and chemical reactions, which improve the soil engineering properties [5]. The curing agents for chemical stabilization of soft soils can be mainly divided into inorganic curing agents and organic curing agents. Among them, inorganic curing agents like Portland cement, fly ash and lime are the most popular and effectively enhance the stability and shear strength of soils [6,7,8,9,10]. In recent years, with the emphasis on resource conservation and environmental protection, more and more industrial and agricultural wastes have been used for soil stabilization, such as wood industry ash, coconut shell ash, rice husk ash (RHA), etc. [11,12,13,14,15,16,17].

The engineering properties of soft soils are effectively improved by chemical stabilization, which meets the standards of engineering applications. At present, strength and water stability are the two engineering properties of stabilized soils that have been given the most attention, while less attention has been paid to deformation performance [18]. This is because creep behaviors of stabilized soils are often considered negligible. However, there have been a large number of engineering practices showing that soft soils after chemical stabilization still have creep deformation [19]. Such deformation is not only affected by the loading level, but also has connections with time and humidity. Therefore, accurate prediction of creep characteristics of soft soils and stabilized soils is of great significance to engineering applications.

A great number of models have been built to characterize the soils’ creep behavior, including empirical semi-empirical (ESE) models, rheological theory-based models, and elastic–viscoplastic (EVP) models [20]. The empirical semi-empirical models are relatively simple and rely on statistical analysis [21]. Researchers have summarized empirical semi-empirical models based on laboratory or field tests of different soils under different test conditions. The representative ones include the Taylor model, Singh–Mitchell model and Mesri model, etc. [22,23,24]. Since then, many improved creep models have been proposed on the basis of the Singh–Mitchell model and Mesri model. For example, Lin et al. [25] proposed a new creep equation considering the over-consolidation factor of clay. Lu et al. [26] utilized a power function to express the stress–strain relationship and a hyperbola equation to express the strain–time relationship. 

The models based on rheological theory often refer to component models such as the Maxwell model, Burgers model and Xiyuan rheological model [27,28,29]. Among them, the Burgers model is more often used to characterize the soft soil deformation behavior. For example, Rajesh et al. [30] used the Burgers model to characterize the soft soil subgrade when studying the performance of geosynthetic-reinforced railway track system lain on soft clay. Huang et al. [31] proposed an improved Burgers model to characterize the creep deformation behavior of soil. The establishment of an EVP model depends on the theory of elasticity and viscoplasticity. There are many classical EVP models for soils, such as the Perzyna model [32] and the EVP models proposed by Yin and Graham [33,34].

The three types of constitutive models mentioned above have their own advantages and disadvantages [21]. The physical implication about the empirical semi-empirical model is not very clear, but it is readily applicable in engineering practice; the rheological theory-based models find it difficult to reflect the creep deformation of rock and soil under complicated conditions; the EVP model can reflect the three-dimensional stresses of soils more accurately, but is relatively complex. 

What is more, most models are formulated based on saturated soils; less consideration is given to unsaturated characteristics of soft soils. In fact, most soils in the field experience unsaturated conditions. When soft soils become unsaturated, they exhibit different volume, strength and hydraulic properties, and these properties are greatly affected by the degree of saturation. The distinctive volume, strength and hydraulic behaviors of soft soils at unsaturated states make the soils exhibit obvious nonlinear characteristics. These characteristics should be considered consistently and coherently in the process of modeling. In other words, an appropriate creep model should reflect the soil behavior under arbitrary pore water pressure and stress value, that is, the soil behavior under the whole hydraulic path and stress path [35,36].

As discussed, soil is often in an unsaturated state in the field. Therefore, this study aims at developing a Mechanistic-Empirical creep model (MEC) for unsaturated soft and stabilized soils. In this model, it considers the stress dependence and incorporates moisture sensitivity using matric suction and shear strength parameters, which could characterize the long-term creep deformation of these soils under various stress levels and water content conditions by one set of parameters, so as to enable more convenient and accurate predictions of the settlement of the foundation and the subgrade in the soft soil areas.

## 2. Development of Mechanistic-Empirical Creep Model for Unsaturated Soils 

### 2.1. Typical Empirical Semi-Empirical Creep Models

The empirical semi-empirical creep models adopt simple mathematical expressions to describe creep properties of soils. Because of its simplicity and convenient parameter acquisition, it is widely used in engineering practice. Mesri and Godlewski [37] introduced a compression index and proposed the following creep model:

(1) $$e=R_{c}C_{c}\mathrm{lg}(1+\frac{t}{t_{1}})$$

in which:

(2) $$R_{c}=C_{\alpha }/C_{c}$$

where *e* is the void ratio; *t*_1_ is the time of the creep process at a certain time; *C*_α_ is the coefficient of secondary compression; and *C_c_* is the compression index.

In order to better consider the nonlinear creep characteristics, Singh and Mitchell [23] proposed a three-parameter creep model, which is shown in Equation (3):

(3) $$\varepsilon =B_{}e^{\beta \overset{-}{D_{1}}}{\left(\frac{t}{t_{1}}\right)}^{\lambda }$$

where *ε* is the strain at any moment; $\overset{-}{D_{1}}=\frac{\left(\sigma_{1}-\sigma_{3}\right)}{{\left(\sigma_{1}-\sigma_{3}\right)}_{f}}$ is the deviator stress level at *t* = *t*_1_; *σ*_1_ – *σ*_3_ is deviator stress of soil at *t* = *t*_1_; (*σ*_1_ – *σ*_3_)*_f_* is damage deviator stress; and *B*, *β* and *λ* are model parameters. The Singh–Mitchell model can better show the creep behaviors of soils, but it is only suitable for describing the strain–time relationship in the range of 20% to 80% of the deviator stress level at the failure point. Later, Mesri [24] improved the creep model on the basis of the Singh–Mitchell empirical model, which is shown in Equation (4):

(4) $$\varepsilon =\frac{2}{{\left(E_{u}/S_{u}\right)}_{1}}\frac{\overset{-}{D_{1}}}{1-{\left(R_{f}\right)}_{1}\overset{-}{D_{1}}}{\left(\frac{t}{t_{1}}\right)}^{\lambda }$$

where EuSu is the undrained modulus to undrained shear strength ratio; (*R_f_*)_1_ and *λ* are model parameters. When *t* = *t*_1_, Equation (4) can be written as Equation (5):

(5) $$\frac{\varepsilon }{\overset{-}{D_{1}}}={\left(\frac{2}{E_{u}/S_{u}}\right)}_{1}+{\left(R_{f}\right)}_{1}\varepsilon$$

so ${\left(\frac{2}{E_{u}/S_{u}}\right)}_{1}$ and (*R_f_*)_1_ can be obtained directly from the $\frac{\varepsilon }{\overset{-}{D}}-\varepsilon$ graph of *t* = *t*_1_ time, and the intercept is ${\left(\frac{2}{E_{u}/S_{u}}\right)}_{1}$ and the slope is (*R_f_*)_1_; *λ* is the mean slope of lg *ε* − lg *t* curves under different stress levels. The Mesri creep model can characterize the deformation behavior of soils under any shear stress level, not being limited to the range of 20–80%.

Thereafter, Tseng and Lytton [38] proposed a popular empirical model for the deformation behavior of subgrade soils and granular materials in 1989, which is shown in Equation (6):

(6) $$\varepsilon_{p}{}^{}=\varepsilon_{0}^{}e^{-{\left(\rho /N\right)}^{\beta }}$$

where *ε_p_* is the permanent strain of the granular material; *ε*_0_ is the maximum permanent strain; *ρ* is the scale factor; *β* is the shape factor; and *N* is the number of cyclic loads. The Tseng–Lytton model can well describe the deformation behavior of soil materials at a certain stress level, but it cannot consider the variation of the stress level. Thus, Gu et al. [39] improved the model by adding $\sqrt{J_{2}}$ and $\alpha I_{1}+K$ into the Tseng–Lytton model. The stress term $\sqrt{J_{2}}$ indicates the influence of the deviatoric shear stress on the materials: when $\sqrt{J_{2}}$ is higher, the deformation will be higher. The stress term $\alpha I_{1}+K$ represents the influence of the hydrostatic stress on the materials, and it is closely related to shear strength parameters. Such a model is called a Mechanistic-Empirical (ME) model, which is expressed as:

(7) $$\varepsilon_{p}=\varepsilon_{0}e^{-{\left(\rho /N\right)}^{\beta }}{\left(\sqrt{J_{2}}\right)}^{m}{\left(\alpha I_{1}+K\right)}^{n}$$

(8) $$\alpha =\frac{2\sin\phi }{\sqrt{3}(3-\sin\phi )}$$

(9) $$K=\frac{c\cdot 6\cos\phi }{\sqrt{3}(3-\sin\phi )}$$

where $J_{2}$ is second invariant of the deviatoric stress tensor; $I_{1}$ is first invariant of the stress tensor; $\varepsilon_{0}$, ρ, β, m, and n are model coefficients; and c and ϕ are cohesion and friction angle, respectively.

With the alteration of water content, the saturation degree of soil changes, and its basic mechanical properties also change greatly. The fundamental difference between unsaturated soils and saturated soils lies in the addition of a new stress state variable, matric suction. Its mechanical properties are related to this variable. The effect of the matric suction on the soil’s properties is essentially a reflection of the impact of water. Therefore, in order to find out the effect of humidity level on the creep deformation of soils, it is necessary to develop a creep model containing the matric suction [40]. In recent years, some researchers have tried to establish an empirical creep model of unsaturated soils by introducing the matric suction to the existing models. For example, on the basis of the Mesri creep model, Lai et al. [41] established a creep model for unsaturated soils as:

(10) $$\varepsilon (t)={\left(\frac{{\left(\sigma_{1}-\sigma_{3}\right)}_{f}}{E_{u}}\right)}_{1}\frac{\overset{-}{D_{1}}}{1-{\left(R_{f}\right)}_{1}\overset{-}{D_{1}}}{\left(\frac{t}{t_{1}}\right)}^{\lambda }$$

in which

(11) $$R_{f}=a(h_{m}/P_{a})+b$$

Combine Equations (10) and (11) to Equation (12):

(12) $$\varepsilon (t)={\left(\frac{{\left(\sigma_{1}-\sigma_{3}\right)}_{f}}{E_{u}}\right)}_{1}\frac{\overset{-}{D_{1}}}{1-{\left(a(h_{m}/P_{a})+b\right)}_{1}\overset{-}{D_{1}}}{\left(\frac{t}{t_{1}}\right)}^{\lambda }$$

where $P_{a}$ is the atmospheric pressure (101.33 kPa); $h_{m}$ is the matric suction; and a and b are model parameters that can be obtained from the $R_{f}$ versus $h_{m}/P_{a}$ curve. As shown in Equation (11), Lai et al. [41] only established a simple regression relationship to consider the matric suction, and replaced the term of ${\left(R_{f}\right)}_{1}$ in the Mesri model by the matric suction expression. In other words, the improved creep model is inherently still an empirical model, and the fitting parameters a and b are greatly affected by the data samples, so the accuracy of model prediction needs to be improved.

Based on the discussions above, it is seen that in order to establish an empirical semi-empirical model which accurately reflects the creep deformation behavior of unsaturated soils on the basis of classical creep models, the original model should contain terms or parameters that are appropriately related to the matric suction so as to accurately reflect its influence on creep. By comparing the creep models above, it is found that the ME model developed by Gu et al. [39] based on the Tseng–Lytton model is promising. The ME model contains mechanical terms of $\sqrt{J_{2}}$ and $\left(\alpha I_{1}+K\right)$ with clear physical meanings, and $\left(\alpha I_{1}+K\right)$ reflects the effect of the shear strength of the material. Therefore, this paper tries to further develop this ME model by introducing the matric suction to reflect the effect of unsaturated characteristics on the long-term deformation behavior of soils, which is elaborated on next.

### 2.2. Formulation of Mechanistic-Empirical Creep Model 

When the ME model was proposed by Gu et al [39]. it only reflected the multi-stress creep deformation of materials under a certain moisture content, but soils in engineering practice are mostly unsaturated and the moisture content changes frequently. Thus, there is a need to establish a creep model for unsaturated soils which can simultaneously respond to various moisture conditions and multi-stress levels. To this end, based on the ME model, this study introduces the matric suction parameter, and establishes the relationship between the water content and the matric suction through the soil–water characteristic curve (SWCC). The improved model, called the MEC model, is formulated as follows:

(13) $$\varepsilon_{p}(t)=\varepsilon_{0}e^{-{\left(\rho /t\right)}^{\beta }}{\left(\frac{\sqrt{J_{2}}}{p_{a}}\right)}^{m}{\left(\frac{\alpha_{1}I_{1}+K_{1}}{p_{a}}\right)}^{n}{\left(p_{a}\right)}^{-(m+n)}$$

(14) $$\alpha_{1}=\frac{2\sin\phi_{1}{}^{\prime }}{\sqrt{3}(3-\sin\phi_{1}{}^{\prime })}$$

(15) $$K_{1}=\frac{c_{1}{}^{\prime }\cdot 6\cos\phi_{1}{}^{\prime }}{\sqrt{3}(3-\sin\phi_{1}{}^{\prime })}$$

where $c_{1}{}^{\prime }$ and $\phi_{1}{}^{\prime }$ are the cohesion and internal friction angle for unsaturated soil, respectively.

The relationship between the matric suction and shear strength is established as Equation (16) [42]:

(16) $$\tau_{f}=c\prime +\left(\sigma_{n}+\theta fh_{m}\right)\tan\phi^{\prime }$$

where $c^{\prime }$ and $\phi^{\prime }$ are the cohesion and internal friction angle of soil in a saturated state, respectively; θ is the volumetric water content; f is the saturation factor; and $h_{m}$ is the matric suction. 

Equation (17) is used to calculate the saturation factor f:

(17) $$\{\begin{array}{l} \;f=\frac{1}{\theta }\;pF<2\\ \;f=1+\frac{S-85}{15}(\frac{1}{\theta }-1)\\ \;f=1\;pF>3.5 \end{array}\;2\leq pF\leq 3.5$$

The matric suction can be expressed in the form of pressure (Pa), water head (cm), or *pF*. The *pF* is a unit introduced from soil science, which represents the logarithmic value of pore water potential energy in centimeter head. In Equation (17), the *pF* is the number of matric suction expressed in *pF* form. The conversion relationships are as follows: $pF=\log10(h_{m}incmH_{2}O)$; $1020\;cmH_{2}O=10^{5}Pa$.

**Figure 1:**
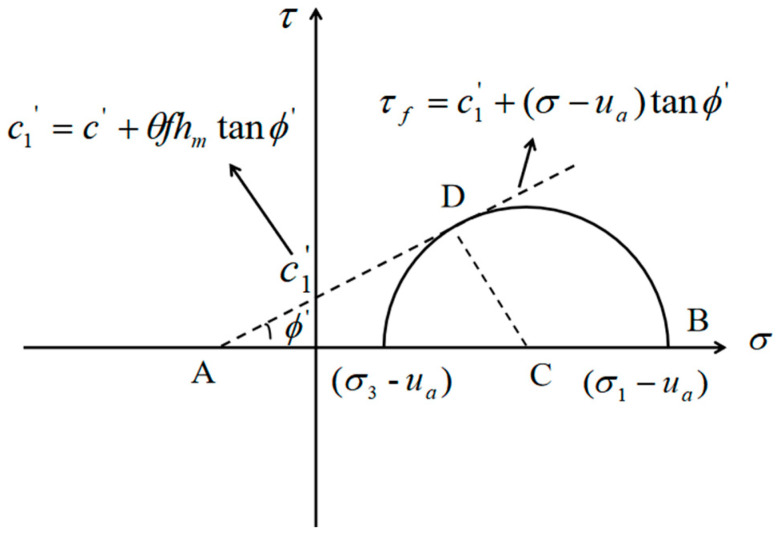
Mohr stress circle expression of failure envelope in two-dimensional plane of net normal stress and shear stress of unsaturated soil.

For unsaturated soils, the apparent cohesion that affects the shear strength of soils includes two terms: one is the conventional cohesion, which represents the shear force produced by the physicochemical interaction between particles such as van der Waals; the second part represents the shear strength produced by capillary action, that is, capillary cohesion, which is mainly related to matric suction in soil [43]. According to the research of Fredlund et al. [44], the influence of matric suction on soil shear deformation is mainly manifested in the suction stress, that is, the effect of capillary cohesion on soil. Fredlund et al. [44] proposed an extended Mohr–Coulomb criterion to represent the shear strength characteristics of unsaturated soils, and found that in the three-dimensional space of stress state variables $\left(\sigma -u_{a}\right)$, $\left(u_{a}-u_{w}\right)$ and shear stress τ, the failure envelope is a planar surface. If the failure plane is projected on the shear stress and net normal stress plane, the expanded Mohr–Coulomb criterion is shown in Figure 1. According to this criterion and Equation (16), the total cohesion of unsaturated soil can be calculated, and is as follows:

(18) $$c_{1}^{\prime }=c^{\prime }+\theta fh_{m}\tan\phi^{\prime }$$

Substitute Equation (18) into Equations (14) and (15), which yields:

(19) $$K_{1}=\frac{c^{\prime }\cdot 6\cos\phi_{1}{}^{\prime }}{\sqrt{3}(3-\sin\phi_{1}{}^{\prime })}+\frac{6\theta fh_{m}\cos\phi_{1}{}^{\prime }\tan\phi^{\prime }}{\sqrt{3}(3-\sin\phi_{1}{}^{\prime })}=K_{n}+K_{m}$$

Therefore, the improved creep model of unsaturated soils is obtained as follows:

(20) $$\varepsilon_{p}(t)=\varepsilon_{0}e^{-{\left(\rho /t\right)}^{\beta }}{\left(\frac{\sqrt{J_{2}}}{p_{a}}\right)}^{m}{\left(\frac{\alpha_{1}I_{1}+K_{n}+K_{m}}{p_{a}}\right)}^{n}{\left(p_{a}\right)}^{-(m+n)}$$

(21) $$\alpha_{1}=\frac{2\sin\phi_{1}{}^{\prime }}{\sqrt{3}(3-\sin\phi_{1}{}^{\prime })}$$

(22) $$K_{n}=\frac{c^{\prime }\cdot 6\cos\phi_{1}{}^{\prime }}{\sqrt{3}(3-\sin\phi_{1}{}^{\prime })}$$

(23) $$K_{m}=\frac{6\theta fh_{m}\cos\phi_{1}{}^{\prime }\tan\phi^{\prime }}{\sqrt{3}(3-\sin\phi_{1}{}^{\prime })}$$

In this model, $J_{2}$ and $I_{1}$ reflect the effect of the stress on creep behavior of soils. The term of $\alpha_{1}I_{1}+K_{n}+K_{m}$ represents the hardening influence of the hydrostatic stress on the soils, which is closely related to these factors, like the material cohesion, internal friction angle, matric suction and moisture status. Among them, $K_{m}$ is a newly introduced item, which is used to further consider the effect of humidity and matric suction on soil deformation. The three moisture-related terms of θ, f and $h_{m}$ are used to compute $K_{m}$.

Through the establishment of the MEC model in Equation (20), it can be used to accurately describe the creep behavior of unsaturated soft soils and unsaturated stabilized soils. Three steps are involved in the process of model application:

(1) Determine the SWCCs of soft and stabilized soils.
(2) Determine the relevant shear strength parameters of soft and stabilized soils.
(3) Determine the coefficients *ε*_0_, *ρ*, *β*, *m* and *n* from the creep tests at different stress levels and moisture conditions.

## 3. Materials and Laboratory Tests

### 3.1. Materials

The test soil is silt clay. Basic physical properties of the soils are list in Table 1. In this study, the soil was remolded after being filtered through a 4.75 mm sieve. 

**Figure 2:**
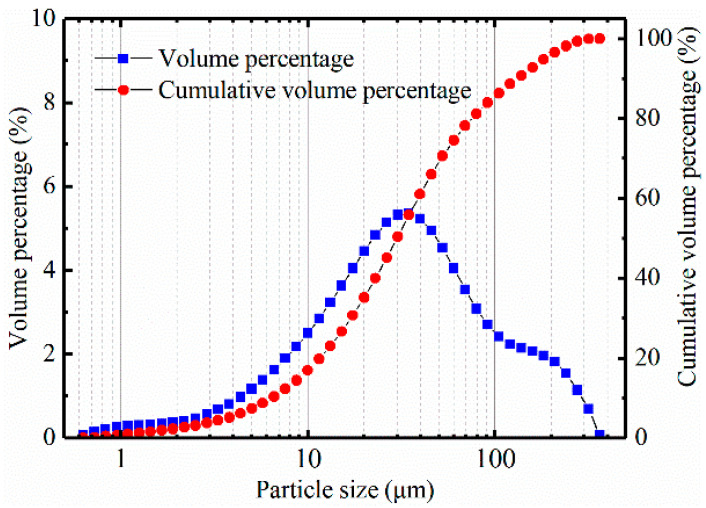
Particle size distribution of RHA.

**Figure 3:**
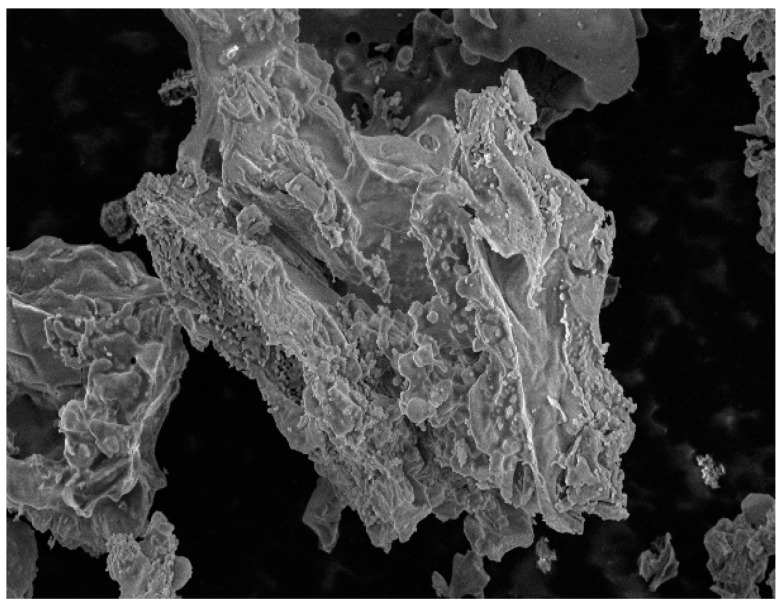
SEM diagram of RHA at 2000 times.

**Figure 4:**
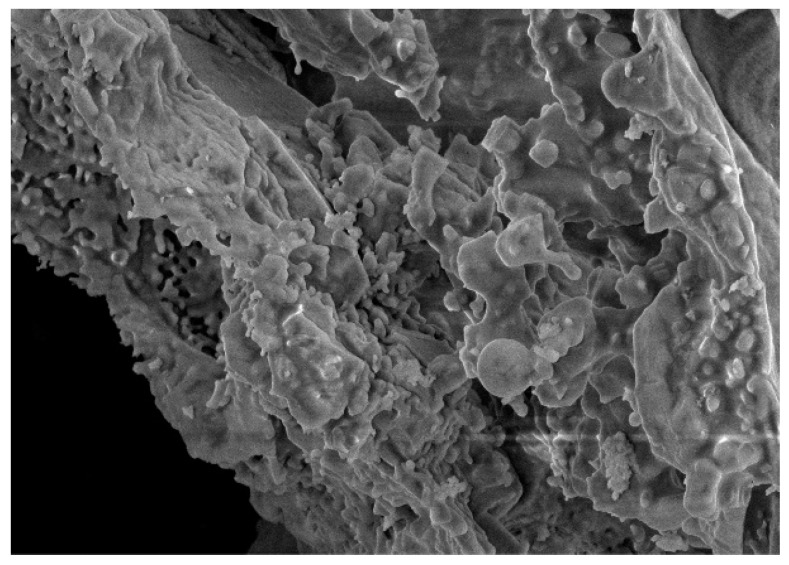
SEM diagram of RHA at 5000 times.

Lime and RHA are used as the stabilization materials in this study. Table 2 shows their chemical compositions. RHA is black powder, and Figure 2 shows the particle size distribution. The microstructure of RHA was studied by scanning electron microscopy (SEM). The magnification was 2000 times and 5000 times, as shown in Figure 3 and Figure 4, respectively. From these figures, it can be seen that RHA contains many fine spherical particles, which are silica.

### 3.2. Test Design

Three types of soil, soft soil (silt clay), lime soil and rice husk ash–lime (RHA–lime) composite stabilized soil, are prepared for testing. The lime content in the lime soil is 5%; the content of lime and RHA in RHA–lime soil is 5% and 4%, respectively. The laboratory tests include a SWCC test, shear strength test, unconfined compressive strength (UCS) test and creep test. The purpose of the SWCC test and shear strength test is to confirm the model parameters $\alpha_{1}$, $K_{n}$ and $K_{m}$, and the UCS test is to determine the stress levels used in the creep test.

### 3.3. Test Methods

#### 3.3.1. Preparation of Specimens

The specimens are remolded at 95% degree of compaction under the optimum moisture contents. The optimum moisture contents were obtained using the light compaction test in the specification of “Test Methods of Soils for Highway Engineering” [45]. The value of the optimum moisture of soft soil, lime soil and RHA–lime soil are 18%, 18.5% and 19%, respectively. The curing age of stabilized specimens is 28 days. The dimension of the specimen for each test is: 70 mm in diameter and 40 mm high for the SWCC test; 61.8 mm in diameter and 20 mm high for the direct shear test; and 70 mm in diameter and 140 mm high for the UCS test and creep test. 

#### 3.3.2. Shear Strength Test

Direct shear test [45] is used to determine the shear strength parameters. Three water contents (15%, 18% and 21%) are selected, and four specimens are tested for each water content. The shear strength test is carried out at 50 kPa, 100 kPa, 150 kPa and 200 kPa stress levels.

#### 3.3.3. Soil–Water Characteristic Curve Test

The test is carried out in accordance with ASTM D5298 [46]. The filter paper and test pieces are put into the sealed tank together and stored at the constant temperature and humidity curing box for 7 days. Finally, the filter paper is weighed with a high-precision balance, and the matric suction of the sample is calculated according to the calibration curve of the filter paper. The calibration equation is according to the matric suction curve equation measured by ASTM:

(24) $$\{\begin{array}{c} \log h_{m}=-0.0673w_{fp}+4.945\begin{array}{cc} & w_{fp}<47\% \end{array}\\ \log h_{m}=-0.0229w_{fp}+2.909\begin{array}{cc} & w_{fp}\geq 47\% \end{array} \end{array}$$

where $h_{m}$ is the matric suction, unit kPa, and $w_{fp}$ is the moisture content when the filter paper is balanced, unit %.

#### 3.3.4. Unconfined Compressive Strength Test and Creep Test

The universal testing machine was used to carry out the UCS test and creep test. The loading rate of the UCS test is kept at 1 mm/min. The creep test adopts the form of a single-stage test, the loading time for each stage is 15,000 s. For each kind of soil, three moisture contents are set, which are 15%, 18% and 21%, respectively. Under the same water content of the same soil, five different stress levels are chosen. The results of the UCS test are used to determine the magnitude of the stress level. The five stress levels are approximately 20%, 40%, 60%, 80% and 95% of the ultimate compressive strength. In the test, an extensometer is used to monitor the deformation of materials. The measurement range is 60 mm in the middle of the specimen (140 mm high).

## 4. Results of Laboratory Soil Tests

### 4.1. SWCC Test Results

**Figure 5:**
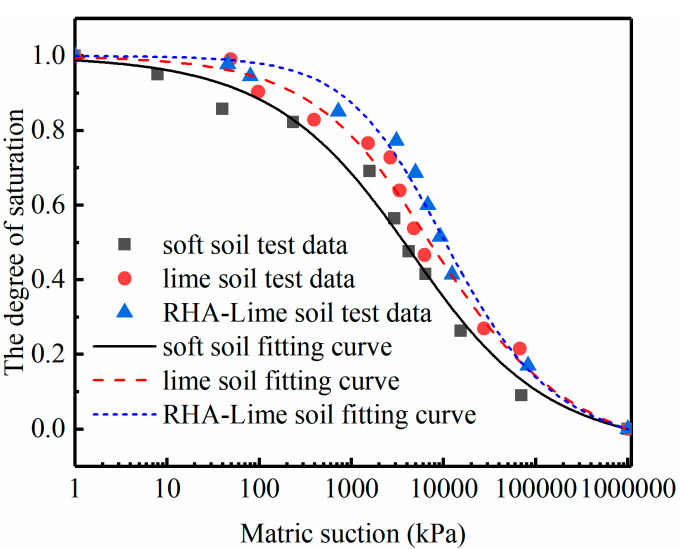
SWCC of the selected three kinds of soils.

After obtaining the matric suction of the three kinds of soils under different water conditions, the Fredlund and Xing model [47] is used to determine the SWCC of each specimen. The Frendlund–Xing model is:

(25) $$S=C(h_{m})\times \left[\frac{1}{{\left\{\ln\left[\exp(1)+{\left(\frac{h_{m}}{a_{f}}\right)}^{b_{f}}\right]\right\}}^{c_{f}}}\right]$$

where $C(h_{m})$ is the correction factor, defined as

(26) $$C(h_{m})=\left[1-\frac{\ln(1+\frac{h_{m}}{h_{r}})}{\ln(1+\frac{10^{6}}{h_{r}})}\right]$$

where *S* is the degree of saturation; $h_{r}$, $a_{f}$, $b_{f}$ and $c_{f}$ are model coefficients. Figure 5 shows the fitting results, and Table 3 [48] shows the values of the model coefficients. 

### 4.2. Shear Strength Test Results

**Figure 6:**
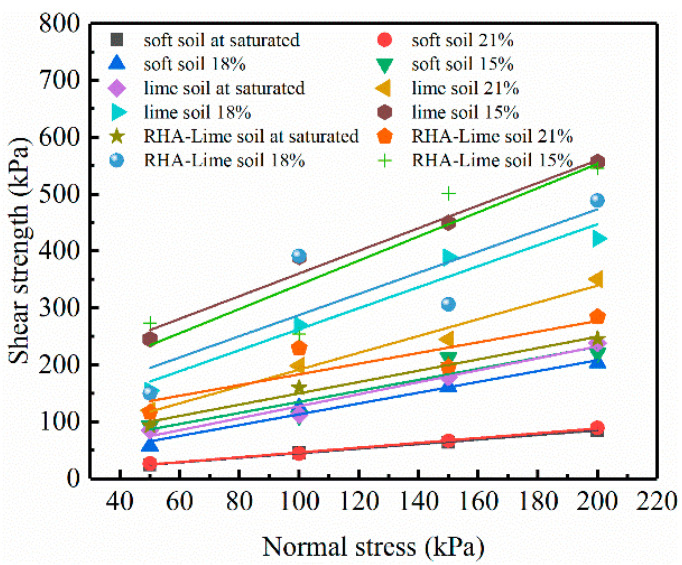
Relationship between shear strength and vertical pressure.

Figure 6 shows the relationship between the shear strength and vertical pressure of each soil type. The corresponding shear strength parameters $c^{\prime }$ and $\phi^{\prime }$ are under a saturated state, and $\phi_{1}{}^{\prime }$ of unsaturated soils under different water contents can be obtained from the test result. 

Based on the results of the SWCC and shear strength test, the matrix suction *h_m_* and shear strength parameters $c^{\prime }$, $\phi^{\prime }$ and $\phi_{1}{}^{\prime }$ can be obtained. The saturation factor *f* can then be obtained according to Equation (17). Finally, the model parameters $\alpha_{1}$, $K_{n}$ and $K_{m}$ of each soil under different water contents can be calculated by substituting the relevant parameters into Equations (21)–(23). The results are presented in Table 4.

### 4.3. Unconfined Compressive Strength Test Results

**Figure 7:**
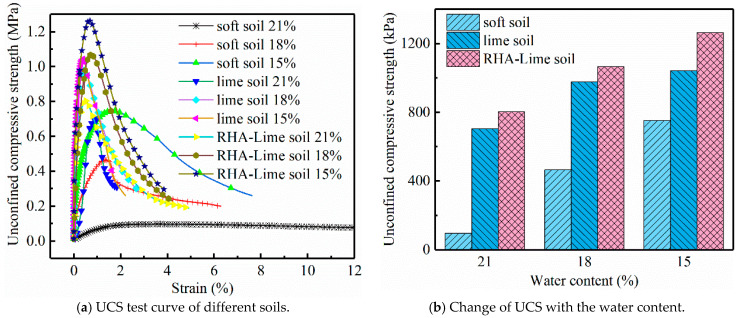
Unconfined compressive strength test results.

**Figure 8:**
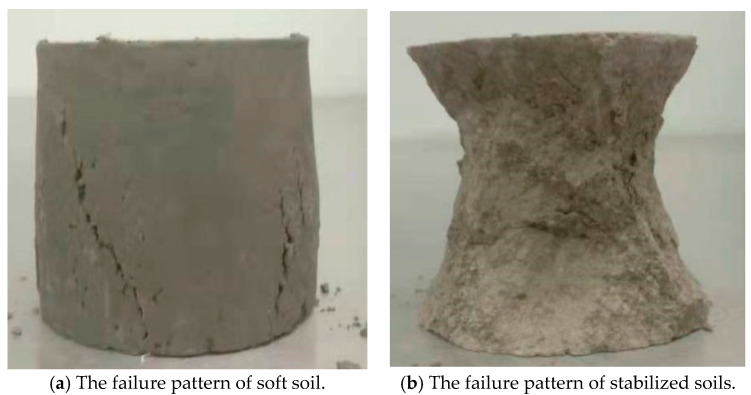
The failure patterns of soils.

Before the creep test, the UCS test should be carried out in order to determine the loading magnitude at each level of the creep test. Figure 7 shows the UCS test curves and the change of the UCS with the water content. Comparisons among the three kinds of soils show that the strength of the RHA–lime soil is highest while that of the soft soil is smallest under the same water content. The effect of the degree of water on UCS of the three kinds of soils is also different. Among them, the soft soils are most affected by the moisture level. The lime soils and RHA–lime soils are less affected and their strength decreases slightly. It can be seen from Figure 8 that the failure patterns of soft and stabilized soils are different. For the soft soil specimen, it presents a lateral swelling failure pattern, while for lime soil and RHA–lime soil, it presents a conical failure pattern. The different failure patterns also make the stress–strain curves of soft soil and stabilized soil different, which can be seen in Figure 7a. With the addition of curing agent, the rising straight line becomes more apparent, and the slope becomes steeper. Meanwhile, the descending section of the stress–strain curve becomes steeper, and the strain becomes smaller during failure. These indicate that the brittleness of soft soil increases after stabilization. The reason for these phenomena is that the admixtures such as lime and RHA to the soil will produce chemical reactions and produce certain cementitious minerals, which will make the soil particles more closely connected and enhance the water stability of the soil. 

## 5. Modeling of Creep Test Results of Unsaturated Soils

The creep tests provide a complete dataset for soft soils, lime soils and RHA–lime soils at three moisture conditions and four to five stress levels. To appraise the accuracy and reliability of the proposed MEC model, this model is applied to all of the testing cases. In addition, two existing creep models are selected for comparative analysis. The two models are the Mesri creep model (denoted as M model) and the improved unsaturated soil creep model proposed on the basis of the Mesri creep model (denoted as IM model). These two empirical semi-empirical creep models are described in Section 2.1 above.

### 5.1. Determination of Creep Model Parameters

The MEC model introduces the stress level term and moisture level term, which can predict the creep deformation behavior well under various water contents and various stress conditions. The MEC model is used to fit the soft soils, lime soils and RHA–lime soils under the conditions of multiple water contents and various stress levels. The fitting process is as follows: according to the results of the SWCC and shear strength tests shown in Table 4 above, the test data points are subjected to multivariate nonlinear fitting analysis using Equation (20). In the fitting analysis, all the test data points of the same soil sample are put together to obtain a set of fitting parameters. For example, for a soft soil sample, there are 15 sets of test data, which are five sets of test data under 21% water content at five stress levels D, five sets of data under 18% water content and five sets under 15% water content. Then the 15 sets of test data are fitted by the MEC model. 

**Figure 9:**
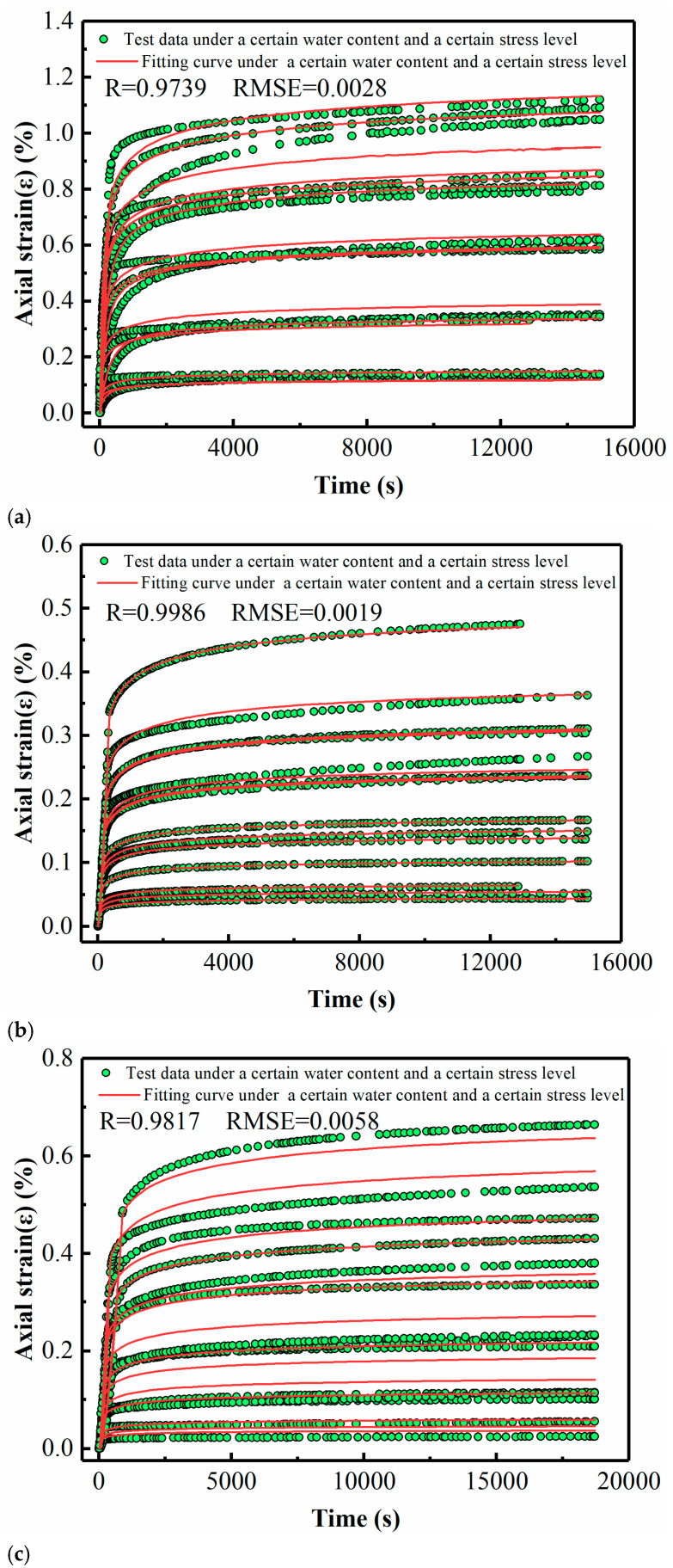
Fitting results of the MEC model under multi-moisture levels. (**a**) Soft soil (including 15 sets of test data, *D* = 0.214, 0.427, 0.646, 0.834 and 0.945 when the water content is 21%, *D* = 0.195, 0.391, 0.587, 0.754 and 0.950 when the water content is 18%, and *D* = 0.172, 0.345, 0.552, 0.759 and 0.966 when the water content is 15%). (**b**) Lime soil (including 14 sets of test data, *D* = 0.184, 0.368, 0.551, 0.735 and 0.919 when the water content is 21%, *D* = 0.160, 0.346, 0.532 and 0.665 when the water content is 18%, and *D* = 0.184, 0.368, 0.551, 0.735, 0.919 when the water content is 15%). (**c**) RHA–lime soil (including 14 sets of test data, *D* = 0.184, 0.368, 0.551, 0.735 and 0.919 when the water content is 21%, *D* = 0.195, 0.391, 0.587, 0.754 and 0.950 when the water content is 18%, and *D* = 0.172, 0.345, 0.552, 0.759 and 0.966 when the water content is 15%).

Figure 9 shows the fitting results, where diagrams a, b and c correspond to soft soil, lime-stabilized soil and RHA–lime-stabilized soil, respectively. The creep test data are shown in the black dots in Figure 9. The MEC model is fitted to the measured data using the software Origin. The fitting curves are shown in the red curves in Figure 9, and the values of the model coefficients are given in Table 5. By comparing the experimental data points and fitting curves in the figure, it can be found that the fitting curves are consistent with the experimental data. The goodness of fitting is demonstrated in Figure 9 with the R-squared values and Root Mean Square Error (RMSE). From the figures, it can be seen that the fitting accuracy of the three soils are very high, with the R-squared values of 0.9739, 0.9986 and 0.9823, respectively.

**Figure 10:**
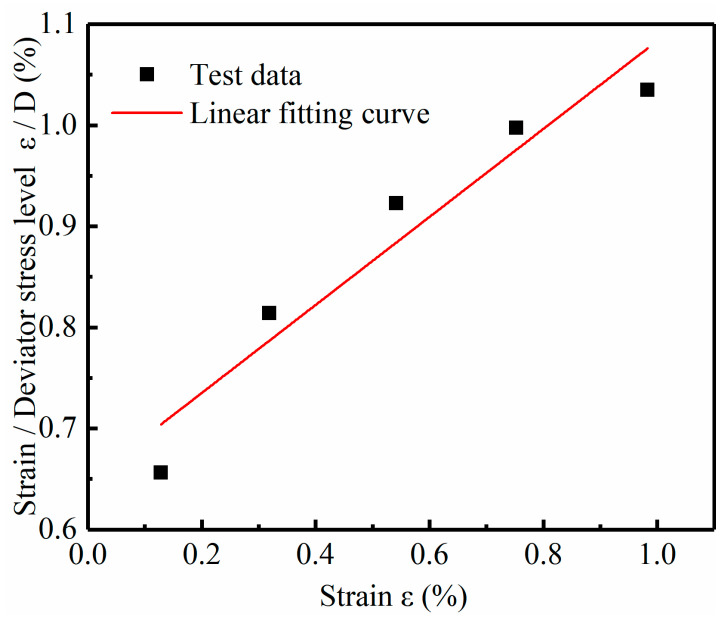
The $\frac{\varepsilon }{\overset{-}{D}}-\varepsilon$ diagram when *t*_1_ = 1 h of soft soil at 18% water content.

**Figure 11:**
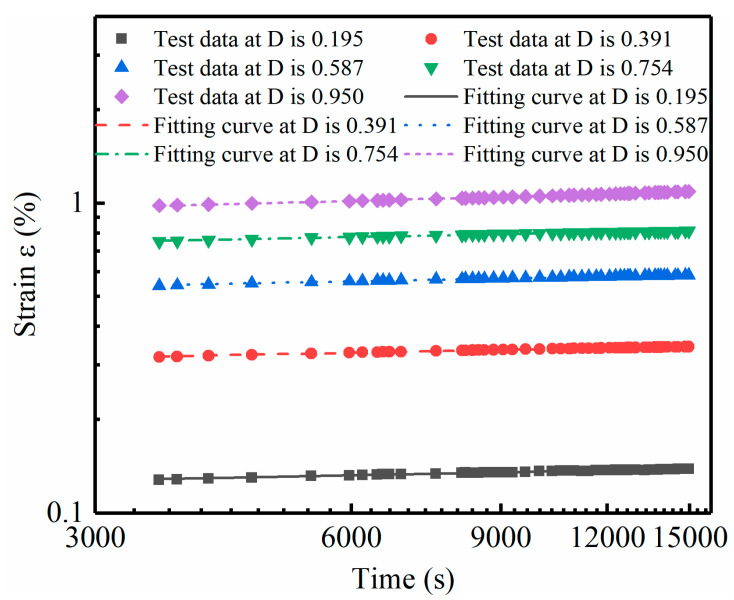
The lg *ε* – lg *t* curve of the soft soil at 18% water content.

**Figure 12:**
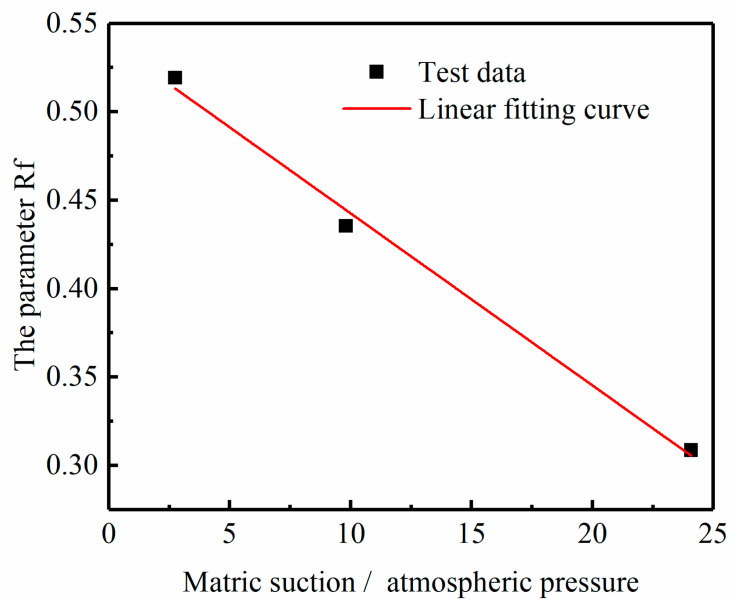
The $R_{f}-\frac{h_{m}}{P_{a}}$ curve of the soft soil.

The parameter-solving processes for the M model and IM model are different from that of MEC model. Its parameters cannot be directly fitted; instead, they need to be solved according to the specific solution method of parameters. The process of solving the parameters of the M model is as follows: take *t*_1_ = 1 h and draw the $\frac{\varepsilon }{\overset{-}{D}}-\varepsilon$ diagram when *t*_1_ = 1 h, as shown in Figure 10, based on which the values ${\left(\frac{2}{E_{u}/S_{u}}\right)}_{1}$ and ${\left(R_{f}\right)}_{1}$ are obtained. Specifically, according to Equation (5), it is known that ${\left(\frac{2}{E_{u}/S_{u}}\right)}_{1}$ and ${\left(R_{f}\right)}_{1}$ are the intercept and slope of the $\frac{\varepsilon }{\overset{-}{D}}-\varepsilon$ curve respectively. Therefore, according to Figure 10, the ${\left(\frac{2}{E_{u}/S_{u}}\right)}_{1}$ and ${\left(R_{f}\right)}_{1}$ values of soft soil under the condition of 18% water content can be calculated. The parameters about ${\left(\frac{2}{E_{u}/S_{u}}\right)}_{1}$ and ${\left(R_{f}\right)}_{1}$ of other soils under different moisture contents are obtained in the same way as above. Then plot the lgε−lgt diagram, as shown in Figure 11, based on which the value of λ is gained. λ is the mean slope of lgε−lgt curves under different stress levels. The process to obtain the parameters for the IM model is similar. The results of ${\left(\frac{2}{E_{u}/S_{u}}\right)}_{1}$ and λ are the same as those in the M model, but the difference is that the parameter ${\left(R_{f}\right)}_{1}$ will be replaced by a and b. According to Equation (11), the relationship between ${\left(R_{f}\right)}_{1}$ and $h_{m}/P_{a}$ can be obtained, from which a and b are the slope and intercept of the curve, respectively. Figure 12 shows the $R_{f}-h_{m}/P_{a}$ curve of soft soil, from which parameters a and b in the IM model of soft soil can be obtained. The method for obtaining a and b parameters of other types of soil is the same as above. Finally, the parameters of these two models are given in Table 5.

### 5.2. Comparison of Different Creep Models

**Figure 13:**
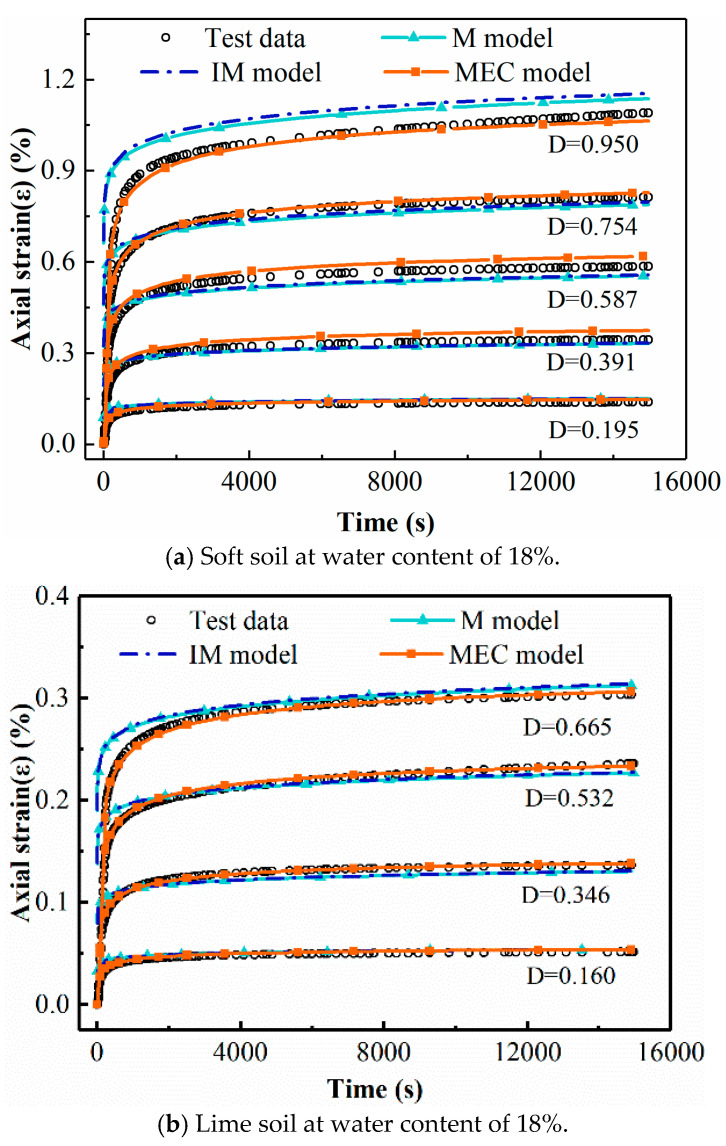

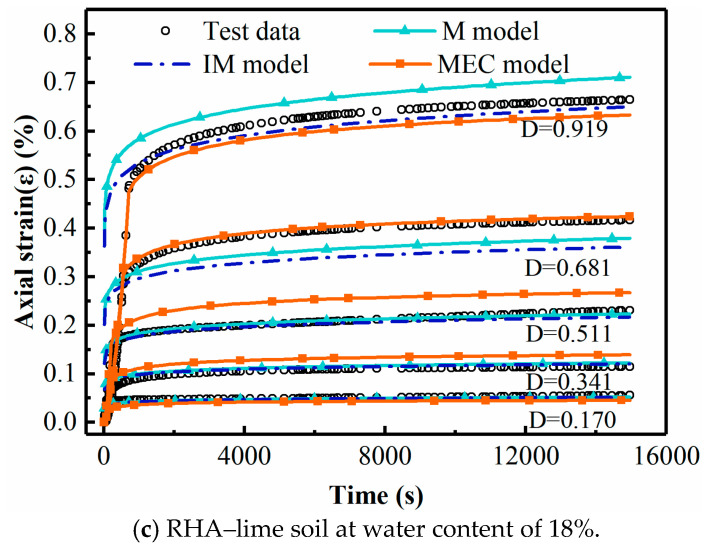
Fitting results of different creep models.

To compare the fitting effects of different creep models, the prediction results of the three models and the test data are plotted in the same graph, with some of the results illustrated in Figure 13. Based on the figures, the prediction results of the three models are in good agreement with the test data, signifying that the MEC model has as good prediction accuracy as the other two classical models. Moreover, it should be emphasized that the most prominent difference between the MEC model and the other two models lies in the ability to predict at multiple moisture conditions by one set of parameters. This conclusion can be directly obtained from Table 5. From Table 5, it can be found that for the same type of soil, the MEC model only has one set of model parameters, while the M and IM models need different sets of parameters at different moisture levels. In other words, once the model parameters are determined, the MEC model could predict the creep deformation behaviors of a soil at any water content. However, for the M and IM models, the process of model determination should be repeated for each water content. For instance, to predict the deformation of soft soil at 10% water content, it is necessary to undertake indoor creep tests and repeat the previous analysis to determine the model parameters at 10% water content when using the M or IM model. However, for the MEC model, the deformation of soft soil with 10% water content can be directly predicted by the MEC model parameters of soft soil in Table 5.

In the process of engineering practice, geotechnical structures such as foundation or subgrade are deformed under the condition of changing stress and moisture, and there is a coupling effect between these two factors. Therefore, in order to aim to predict the creep behavior of soils accurately in engineering practice, the stress and moisture levels need to be considered at the same time. That is to say, an appropriate mode should predict the creep deformation behavior under different water contents and different stress conditions. It is obvious that the MEC model is more convenient and suitable for this purpose. That is to say, compared to the other two classical models, the MEC model not only has good prediction accuracy, but also has greater convenience and practicability. It can be easily used to predict the deformation of unsaturated soil under any stress level and water content condition.

## 6. Model Implementation for Predicting Subgrade Deformation

To analyze the deformation of the three types of soil under various water content conditions, in the finite element analysis, the MEC model is implemented to compute the creep deformation of a typical flexible pavement structure through a simplifying method [39,49]. In this way, the curing effect of the two kinds of solidification materials can be visualized, which provides a certain reference for the material selection of engineering in a soft soil area. The specific process and analysis results are as follows.

**Figure 14:**
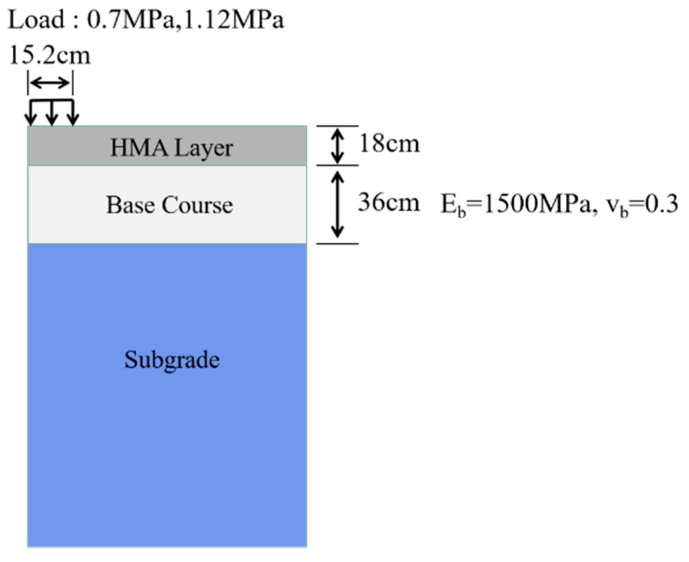
Illustration of pavement structure model and corresponding parameters.

The pavement structure model is shown in Figure 14. The viscoelastic model is used for the surface layer and the elastic model is employed for the other layers. Among them, the resilient modulus of the subgrade is calculated according to the approximate formula (27) between UCS and resilient modulus of the subgrade [50].

(27) $$M_{r}(\mathrm{MPa})=0.124q_{u}(\mathrm{kPa})+68.8$$

where $M_{r}$ is resilient modulus of subgrade; $q_{u}$ is UCS.

**Figure 15:**
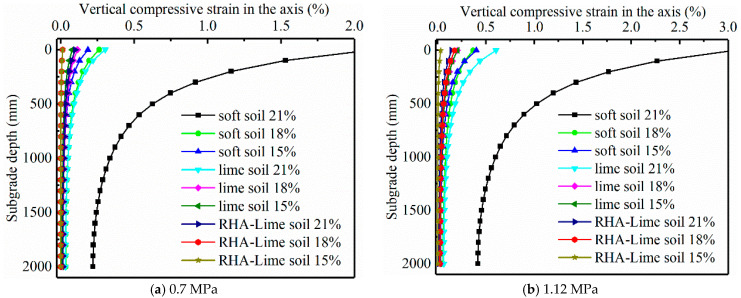
Distribution of vertical compressive creep strain in the subgrade.

In the FE analysis, two common traffic loading levels are selected as 0.7 MPa and 1.12 MPa [51] to compute the stress distributions of the subgrade. Then, the MEC model is applied to calculate the vertical compressive creep strain at corresponding locations for one year. Figure 15 presents the distributions of the creep strains of soft and stabilized soils at different moisture levels. Then the total creep deformation was computed by the multi-layered incremental approach, as shown in Equation (28).

(28) $$\delta_{s}(t)=\int_{0}^{h}\varepsilon_{0}e^{-{\left(\rho /t\right)}^{\beta }}{\left(\frac{\sqrt{J_{2}}}{p_{a}}\right)}^{m}{\left(\frac{\alpha I_{1}+K+K_{m}}{p_{a}}\right)}^{n}{\left(p_{a}\right)}^{-(m+n)}dz$$

where $\delta_{s}$ is the creep deformations of subgrade; *t* is the creep time; and *h* is depth of the effective deforming zone of the subgrade.

**Figure 16:**
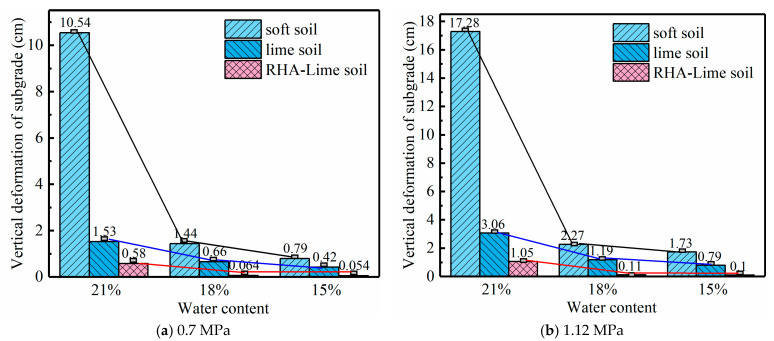
The creep deformation of the subgrade in the vertical direction.

The depth of the effective deforming zone of a soil foundation is set as the location where the stress is 1/10 of the gravity stress [52]. The creep deformations of the three types of soils under different stress conditions with different water contents are obtained. Figure 16 present the results.

From Figure 16, it can be seen that under the same load and moisture level, the deformation is from small to large, in the order of RHA–lime-stabilized soil, lime soil and soft soil. For the same kind of soil, water content has a great influence on its deformation, and the deformation in the wet state is much larger than that in the dry state. The deformation of soft soils is most affected by the moisture level. Through the analysis results of two stress levels, it can be seen that the deformation of the subgrade under a heavy load is obviously larger than that under standard vehicle load. In addition, the deformation of the soft soils is much larger than that of stabilized soil when the same load is applied. Among the three types of soils, the RHA–lime composite soils deform least. In other words, the creep deformation of the soft soil subgrade can be effectively reduced by chemical stabilization, and the effect of RHA–lime composite stabilization is better than that of only adding lime. The reason for these phenomena is that the addition of the curing agent changes the soil structure, which make the deformation behaviors of the soil change.

## 7. Conclusions

This study proposed an improved creep model and validated for soft and stabilized soils, which can characterize the deformation behavior of unsaturated soils very well. The main findings of this work are as follows:

(1) The MEC model takes into account the stress dependence based on mechanical principles, and incorporates moisture sensitivity using matric suction and shear strength parameters. This formulation is intended to characterize the creep deformation behavior of unsaturated soils under arbitrary water content and arbitrary stress condition.
(2) The deformation of unsaturated soils was analyzed by the MEC model under various stress and moisture conditions. The results show that the predicted results of the MEC model are consistent with the experimental data with very high R-squared values.
(3) Compared with the classical unsaturated soil creep models, the MEC model only needs one set of parameters for different stress levels and moisture conditions, while the classical models (like the Mesri and improved Mesri models) require a different set of parameters when the water content is changed. In addition, the MEC model agrees with the experimental data better for stabilized soils, and provides better accuracy in predicting creep deformations at high stress levels.
(4) In the FE analysis, the MEC model is implemented to analyze the creep behavior of subgrade soils. Loading level and moisture have a great effect on the deformation of soil foundation, especially soft soils. Under heavy loading and a wet state, the deformation of soft soil increases rapidly.
(5) After stabilization, the deformation of the soil foundation is obviously reduced. Under the same load and moisture level, the deformation of soft soil is largest, followed by lime soil and RHA–lime-stabilized soil, respectively.

## Figures and Tables

**Table 1:** Basic physical properties of soil samples.

Soil Type	Liquid Limit, WL/%	Plastic Limit, WP/%	Plastic Index, IP	Optimum Moisture Content/%	Maximum Dry Density/(kg/m^3^)
Silt clay	38	19	19	18	1798

**Table 2:** Chemical composition of stabilized materials.

Materials	SiO_2_	CaO	Al_2_O_3_	MgO	Others
Lime	-	86.2	-	0.68	-
RHA	88.09	0.98	1.25	0.34	-

**Table 3:** Results of the SWCC model coefficients.

Materials	*h_r_*	*a_f_*	*b_f_*	*c_f_*
Soft soil	3000	2000	0.509	1.581
Lime soil	3000	3059	0.589	1.192
RHA–lime soil	3000	7980	0.787	1.241

**Table 4:** Values of the parameters in the MEC model.

Materials	*w* (%)	*θ* (%)	*f*	*h_m_* (kPa)	$c^{\prime }\;(\mathrm{kPa})$	$\phi^{\prime }\;({}^{\circ})$	$\phi_{1}^{\prime }\;({}^{\circ})$	*α*_1_	*K_n_*	*K_m_*
Soft soil	21	36.4	1	124	3.885	21.25	22.88	0.172	4.75	20.92
	18	31.2	1	993	3.885	21.25	43.54	0.344	4.22	130.86
	15	26.0	1	2439	3.885	21.25	44.11	0.349	4.20	266.14
Lime soil	21	36.7	2.11	71	52.105	46.54	47.00	0.372	54.28	60.39
	18	31.5	1	723	52.105	46.54	55.90	0.440	46.62	214.85
	15	26.2	1	2276	52.105	46.54	61.54	0.479	40.59	489.82
RHA–lime soil	21	36.0	1	504	62.57	44.98	53.21	0.420	59.03	171.04
	18	30.9	1	2214	62.57	44.98	61.73	0.480	48.48	529.32
	15	25.8	1	5995	62.57	44.98	64.80	0.498	44.10	1088.41

**Table 5:** Values of the parameters of different creep models.

	MEC Model Parameters					M Model Parameters			IM Model Parameters			
	*ε*_0_	*ρ*	*β*	*m*	*n*	${\left(\frac{2}{E_{u}/S_{u}}\right)}_{1}$	${\left(R_{f}\right)}_{1}$	*λ*	${\left(\frac{2}{E_{u}/S_{u}}\right)}_{1}$	*λ*	*a*	*b*
Soft soil 21%	5.948	0.012	0.318	1.706	−1.327	0.523	0.519	0.084	0.523	0.084	−0.010	0.540
Soft soil 18%						0.648	0.435	0.056	0.276	0.056		
Soft soil 15%						0.751	0.309	0.066	0.751	0.066		
Lime soil 21%	19.047	0.012	0.340	1.225	−0.021	0.169	0.576	0.061	0.169	0.061	−0.008	0.572
Lime soil 18%						0.289	0.509	0.052	0.289	0.052		
Lime soil 15%						0.326	0.391	0.079	0.326	0.079		
RHA–lime soil 21%	58.449	0.013	0.311	1.674	−0.253	0.158	0.868	0.051	0.158	0.051	−0.009	0.938
RHA–lime soil 18%						0.239	0.768	0.073	0.239	0.073		
RHA–lime soil 15%						0.478	0.374	0.083	0.478	0.083		

## Data Availability

Not applicable.
